# Long term survival of patients with alpha-fetoprotein-positive hepatoid adenocarcinoma of the gallbladder: a case report and literature review

**DOI:** 10.3389/fonc.2026.1679309

**Published:** 2026-06-30

**Authors:** Fangzhou Wang, Xiang Gao, Wei Gao, Jie Cai, Yamin Zheng

**Affiliations:** 1 Department of General Surgery, Xuanwu Hospital, Capital Medical University, Beijing, China; 2 Department of Pathology, Xuanwu Hospital, Capital Medical University, Beijing, China; 3 Health Management Department, Xuanwu Hospital, Capital Medical University, Beijing, China

**Keywords:** AFP, case report, gallbladder, gallbladder carcinoma, hepatoid adenocarcinoma

## Abstract

Hepatoid adenocarcinoma (HAC) of the gallbladder is an exceedingly rare and highly aggressive variant of gallbladder carcinoma that is frequently associated with alpha-fetoprotein (AFP) production and typically has a poor prognosis. We report a 66-year-old male with a two-week history of right upper abdominal pain. Imaging revealed a hypermetabolic mass at the gallbladder fundus, accompanied by a significantly elevated serum AFP concentration of 1210.00 ng/mL. Histopathological and immunohistochemical analyses confirmed a diagnosis of AFP-positive HAC. Notably, next-generation sequencing (NGS) revealed a *TP53* mutation and *EGFR* copy number amplification, providing insights into the molecular landscape of this rare tumour. The patient underwent radical resection, including cholecystectomy and wedge hepatectomy. To target the hepatoid biological features of the tumour, adjuvant transarterial chemoembolization (TACE) with epirubicin was administered one month after surgery. Following this combined approach, the patient’s serum AFP concentration normalized. As of January 2025, the patient has achieved a 68-month recurrence-free survival. This case highlights that aggressive surgical intervention combined with adjunctive TACE may significantly improve long-term outcomes in patients with AFP-positive gallbladder HAC.

## Introduction

Gallbladder carcinoma (GBC) is a prevalent malignancy of the biliary tract with a poor prognosis and low overall survival rates. Approximately 90% of GBCs are histologically classified as adenocarcinomas. AFP-producing gallbladder carcinoma is a rare subtype of which is identified as HAC. Elevated AFP is a hallmark of both HAC and clear cell carcinoma, both of which are extremely rare. The former usually consists of large polygonal cells resembling hepatocellular carcinomas, whereas the latter presents with well-differentiated papillary or tubular structures lacking hepatoid features. Occasionally, a combination of both variants is observed.

Historical milestones include Ikeda’s 1992 report of a moderately differentiated AFP-positive gallbladder adenocarcinoma (1), followed by Watanabe’s 1993 description of the first case, exhibiting both elevated AFP and hepatoid differentiation (2). Vardaman subsequently provided the first detailed characterization of gallbladder HAC in 1995 (3). Given the rarity, complex histology, and typically poor outcomes of AFP-producing GBC, we present a case of AFP-positive gallbladder HAC with exceptional long-term survival, along with a comprehensive review of the literature.

## Case presentation

**Figure 1:**
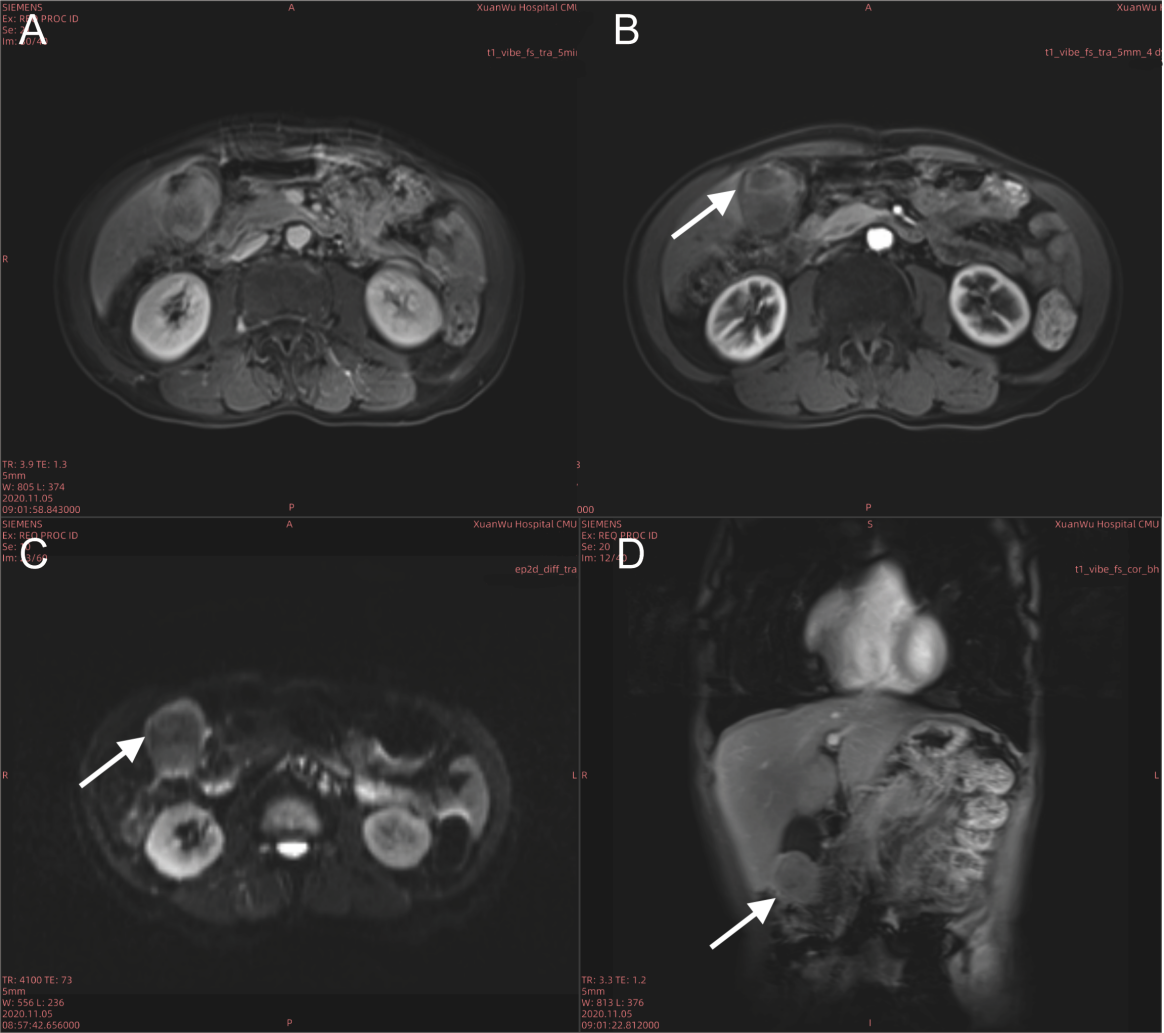
Representative MR findings of the hepatoid adenocarcinoma. **(A)** Axial MR image showing a round abnormal signal at the gallbladder fundus. **(B, D)** Axial and coronal contrast-enhanced MRI in the arterial phase showing early enhancement of the mass (white arrows), mimicking the hemodynamic pattern of hepatocellular carcinoma. **(C)** Diffusion-weighted image showing restricted diffusion, reflecting the aggressive nature of the lesion.

**Figure 2:**
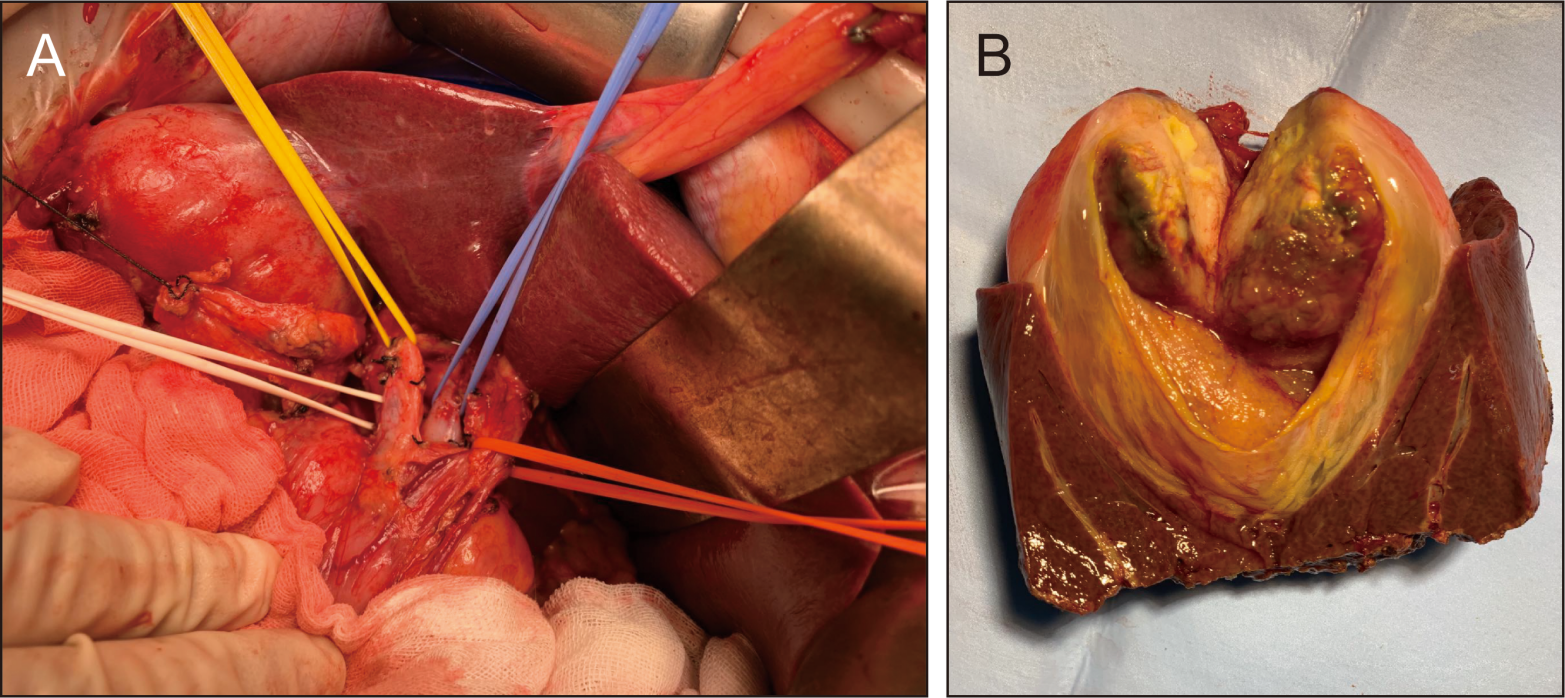
Intraoperative surgical field and macroscopic specimen examination. **(A)** Intraoperative view during radical resection of gallbladder carcinoma, including cholecystectomy combined with wedge hepatectomy. Key anatomical structures are identified and protected using coloured vessel loops: hepatic artery (red), portal vein (blue), and common bile duct (yellow). **(B)** Macroscopic appearance of the resected gallbladder. Longitudinal section revealing a firm, grayish-white and yellowish polypoid mass (arrow) within the gallbladder lumen.

In November 2020, a 66-year-old male with a 30-year history of hepatitis B virus (HBV) infection presented with right upper abdominal pain for two weeks’ duration. Physical examination on admission was unremarkable, with no palpable abdominal masses, tenderness, or jaundice. Laboratory evaluations revealed a significantly elevated serum AFP concentration of 1210.00 ng/ml, whereas carcinoembryonic antigen (CEA), CA72-4, and CA19–9 levels were within normal limits. Preoperative imaging, including ultrasound, magnetic resonance imaging (MRI), and positron emission tomography-computed tomography (PET-CT), revealed a 4.3 × 3.1 cm hypermetabolic, polypoid mass at the gallbladder fundus (SUVmax: 5.24). On MRI, the lesion was round, showing a slightly low signal on T1-weighted images and a slightly high signal on T2-weighted images (**Figure 1**). No lymphadenopathy or distant metastasis was detected. The patient underwent radical resection of the gallbladder carcinoma on November 13, 2020 (**Figure 2**).

**Figure 3:**
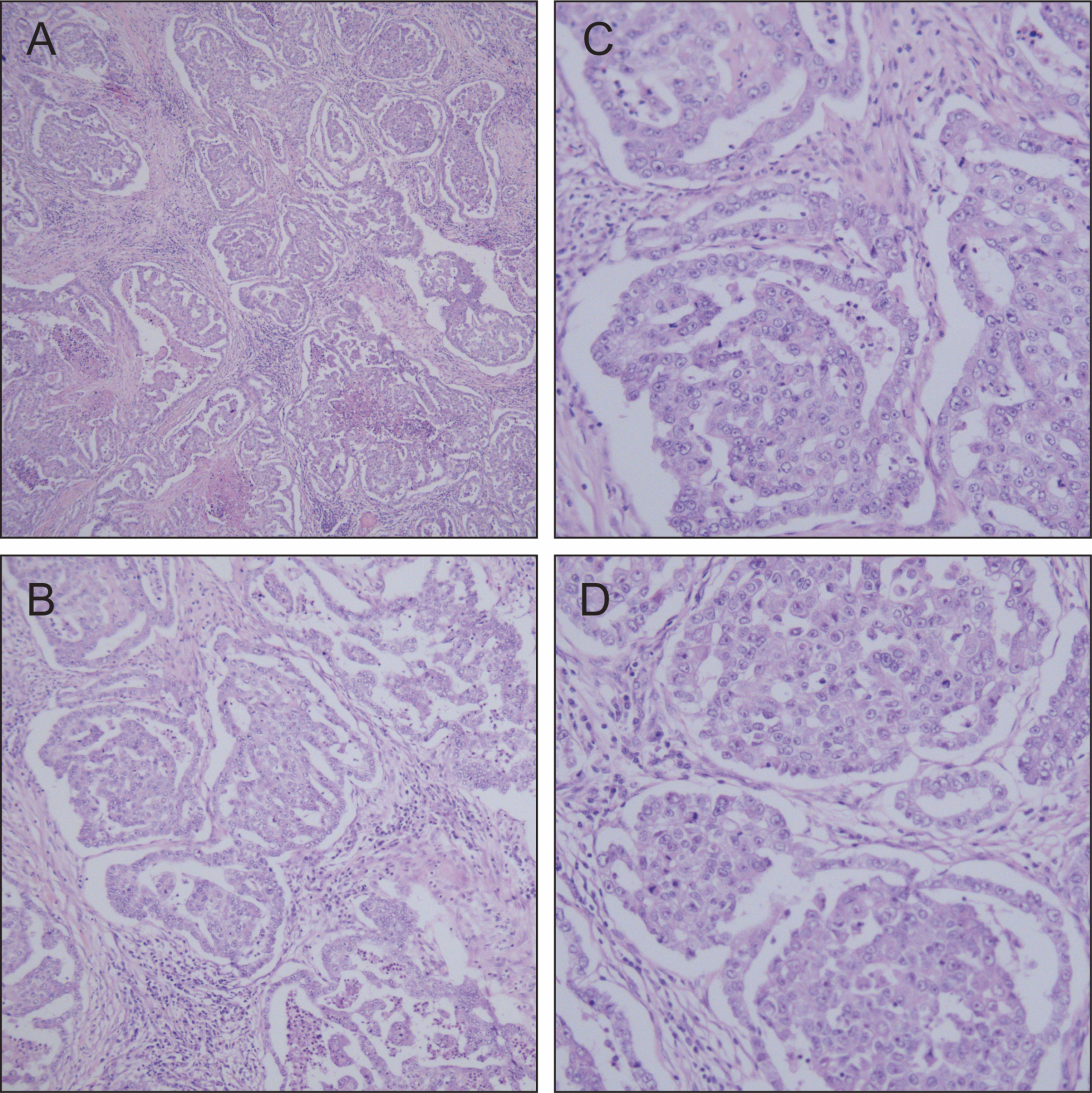
Histopathological characteristics of gallbladder hepatoid adenocarcinoma. **(A)** Low-magnification (×40) hematoxylin and eosin staining showing the overall nested and trabecular architecture of the tumour mass. **(B)** Representative section (×100) demonstrating regions of intraepithelial neoplasia within the gallbladder mucosa. **(C, D)** High-magnification views highlighting characteristic hepatoid differentiation: large polygonal tumour cells (arrows) exhibiting centralized nuclei, prominent nucleoli, and abundant eosinophilic, glycogen-rich cytoplasm.

**Figure 4:**
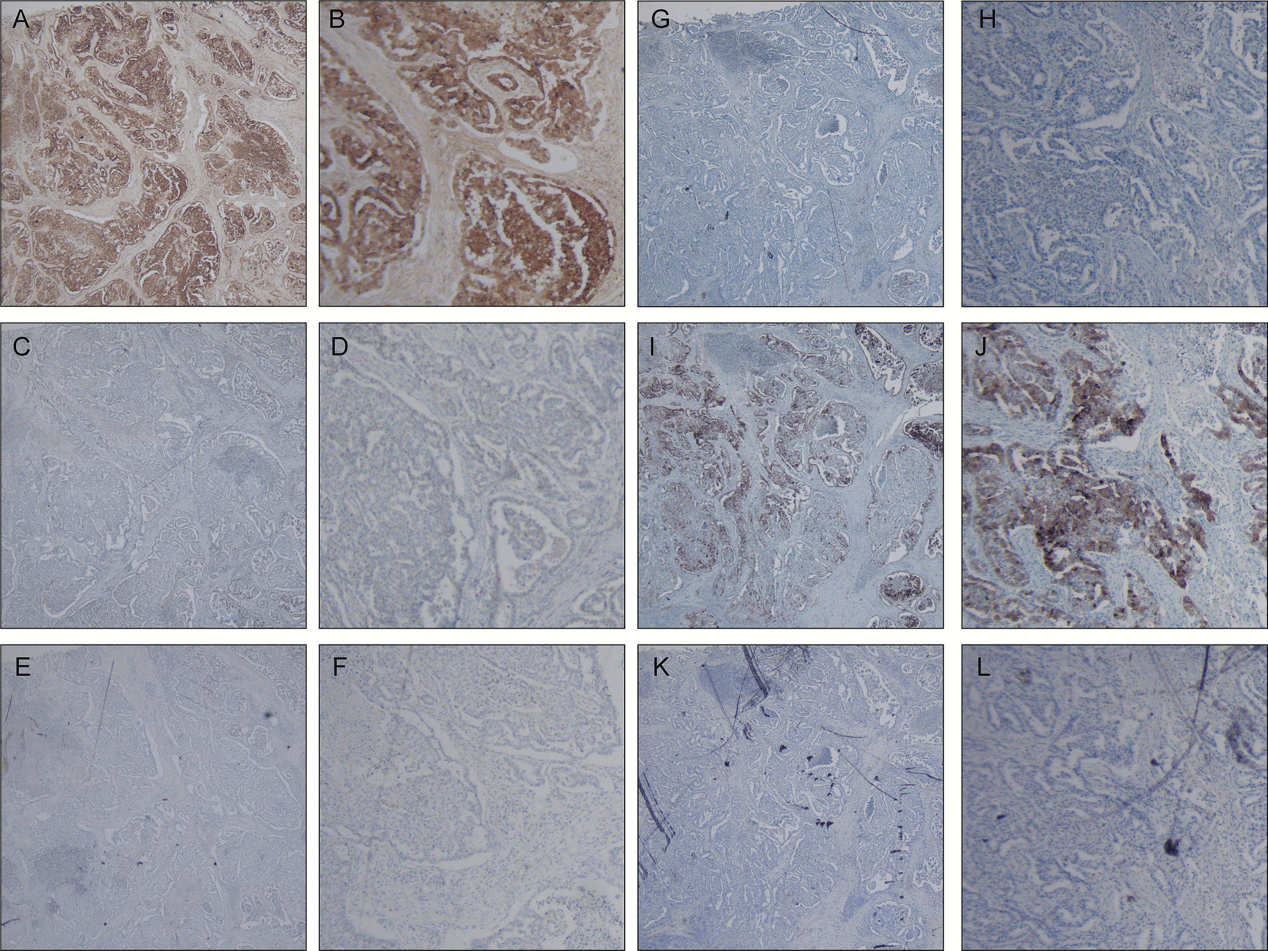
Immunohistochemical staining profile of the gallbladder tumor. Representative microscopic images are presented in pairs, with low-magnification (× 25) images in the left columns and high-magnification (× 40) images in the right columns. **(A, B)** Strong cytoplasmic positivity for AFP within the hepatoid components. **(C, D)** Partial nuclear positivity for SALL-4. **(E, F)** Immunostaining for CK7. **(G, H)** Negative expression of ARG-1. **(I, J)** Significant cytoplasmic and membranous positivity for GPC-3. **(K, L)** Negative staining for Hep Par-1.

Histopathological analysis revealed a moderately differentiated gallbladder adenocarcinoma with papillary regions and focal intravascular tumour thrombi. Immunohistochemical (IHC) staining showed strong positivity for AFP, GPC-3, CK18, and CK19, as well as partial positivity for SALL-4, confirming the diagnosis of HAC. The Ki-67 index was 70%, while the Hep Par-1 and ARG-1 indices were negative (**Figures 3**, **4**). NGS-based genomic profiling identified a *TP53* p.P71Tfs52 mutation (9.96% abundance) and an increase in the number of *EGFR* copies (12.0% abundance). Other alterations included *ABCB11* (p.E283, 3.07%), *ARID1B* (p.Q128_Q131del, 3.70%), *CREBBP* (p.L2087Q, 16.67%), *DROSHA* (p.P95L, 17.69%), and *KCNJ5* (p.Q192*, 5.19%). The tumour mutational burden was low (4.47 Muts/Mb), and the microsatellite status was stable.

Postoperatively, the patient’s serum AFP concertation decreased steadily to 822.60 ng/mL (Day 4), 435.00 ng/mL (Day 11), and 17.00 ng/mL (Day 39). One month after surgery, the patient received adjuvant TACE (30 mg epirubicin), targeting the tumour’s hepatoid biological features. During the 68-month follow-up period, the patient remained free of recurrence or metastasis. As of January 2025, his serum AFP concentration remained stable at 1.74 ng/mL.

## Discussion

Gallbladder HAC is a rare and aggressive histological subtype that mimics HCC morphologically and immunophenotypically. This case is particularly significant because of the patient’s 68-month survival, which far exceeds the typical prognosis reported in the literature (4).

AFP-producing GBCs are heterogeneous. While HAC is the most distinct subtype, defined by a trabecular architecture and abundant eosinophilic cytoplasm, AFP production can also occur in conventional adenocarcinomas or mucinous carcinomas (5, 6). As summarized in **Table 1** (4–14), our literature review includes patients with either elevated serum AFP levels or hepatoid morphology, regardless of their IHC AFP status. These findings suggests that AFP expression may reflect aberrant differentiation pathways or oncofetal protein reactivation, rather than a strict lineage. In this case, the combination of high serum concentration (1210.00 ng/mL) and positive results on IHC staining for AFP, GPC-3, and SALL-4 provided a definitive diagnosis.

**Table 1:** Clinicopathological characteristics of AFP-producing gallbladder carcinoma.

Year	Sex/Age	symptoms	Imaging findings	Tumor markers	Microscopic appearance	Immuno-histochemistry	Outcome	Survival (months)	Ref.
2000	Male/77	Fatigue, fever, loss of appetite and weight	Gallbladder mass and metastases in liver segments IVa and V	AFP: 3.5 ng/ml; CEA: 0.8 ng/ml;	Tumor cells with eosinophilic cytoplasm, enlarged nuclei and prominent nucleoli arranged in nests	CK7, CK8 and CK18 positive. CK19, AFP, CEA and CA19–9 negative	Alive	15	(7)
2004	Male/72	Abdominal pain	Thickened gallbladder wall and liver metastases	CA 19-9: 42.0 U/ml	Tumor cells with eosinophilic cytoplasm, enlarged nuclei and prominent nucleoli arranged in trabeculae	CK8, CK19 and HepPar-1 positive. CK17, AFP and CEA negative	Dead	5	(5)
2005	Female/74	Incidental finding	Gallbladder mass and gallstones	Not reported	Tumor cells with eosinophilic cytoplasm, enlarged nuclei and nucleoli arranged in trabeculae	CK8, CK19, AFP, HepPar-1 positive. CK7 negative	Not reported	/	(8)
2005	Female/74	Incidental finding	Gallbladder mass	Not reported	Tumor cells with eosinophilic cytoplasm, enlarged nuclei and nucleoli arranged in trabeculae	CK8, CK19, HepPar-1 and CD10 positive. CK7 and AFP negative	Not reported	/	(8)
2005	Female/71	Abdominal pain	Gallbladder mass and acute cholecystitis	Not reported	Polygonal tumor cells with eosinophilic cytoplasm, enlarged nuclei and prominent nucleoli arranged in trabeculae	AFP, Victoria blue, Stein and PAS positive.	Dead	1	(14)
2007	Female/76	Abdominal pain, fatigue, loss of appetite	Thickened gallbladder wall	Not reported	Polygonal tumor cells with eosinophilic cytoplasm, enlarged nuclei and prominent nucleoli arranged in trabeculae	HepPar-1, AFP and pCEA positive. CK7 negative	Alive	8	(9)
2007	Female/55	Abdominal pain	Gallbladder mass and liver metastases	AFP: 511000 ng/ml;	Hepatocyte-like adenostructures	CK7, CK19, AFP, CD10 and CD56 positive. CK20 negative	Not reported	/	(10)
2011	Male/61	Incidental finding	Liver metastases after gallbladder surgery	AFP: 967.5 ng/ml;	Highly pleomorphic cells arranged in a rosettoid, nested, or trabecular pattern	AFP positive, HepPar-1, CK7 and CK20 negative	Dead	5	(11)
2015	Femalt/43	Abdominal pain	Gallbladder bed with high signal intensity in the periphery of gall bladder	AFP: 23500 ng/ml;	Adjacent infiltrating neoplasm composed of polygonal cells arranged in papillae, sheets and trabecular pattern	HepPar-1, AFP and CEA positive. CK7 and CK20 negative	Dead	1	(12)
2018	Female/46	Abdominal pain	Thickened gallbladder wall and liver metastases	AFP: 4068 ng/ml;	Well-differentiated adenocarcinoma with papillary configuration	AFP, CK7, Hepatocyte positive, CK20 negative	Not reported	/	(4)
2020	Male/61	Incidental finding	A mass in gallbladder bed with enlarged hilar lymph nodes.	CEA: 104.7 ng/ml, CA12-5 421.3U/ml, AFP: normal range.	Poorly differentiated cells with abundant eosinophilic cytoplasm.	CK7 and HepPar-1 positive	Alive	5	(13)
2022	Female/69	distention of the hypogastrium	Soft tissue mass shadow in the neck of the gallbladder	CEA, AFP, CA19–9 normal range	cuboidal or polygonal with abundant eosinophilic granular hepatocyte-like neoplastic cells	CK7, CK8, HarPar-1, GPC3 and Muc1 positive, AFP negative.	Alive	Not reported	(6)

The genomic landscape of gallbladder HAC remains largely unexplored. The identification of a *TP53* mutation and *EGFR* copy number amplification underscores the profound molecular complexity and aggressive nature of this subtype. As *TP53* alterations are recognized as central drivers in the molecular carcinogenesis of various cancers, their presence in this case likely facilitated genomic instability and tumor progression (15). Concurrently, EGFR amplification indicates a potential therapeutic vulnerability (16); such findings suggest a theoretical pathway for targeted interventions in refractory cases where conventional gemcitabine-based regimens may fail. Beyond these primary drivers, the detection of specific mutations in *ABCB11*, *ARID1B*, and *CREBBP* provides further insight into the unique biology of tumours. The *ABCB11* gene is critical for bile acid transport, and its mutation may be mechanistically linked to the aberrant differentiation of pluripotent endodermal stem cells into hepatoid-like lineages (17, 18). Furthermore, *ARID1B* and *CREBBP* function as essential epigenetic regulators; their involvement points to dysregulated chromatin remodelling, a hallmark of aggressive malignancies (19, 20). Although the tumour mutation burden was relatively low and PD-L1 expression was negative, these integrated genomic findings establish a preliminary molecular map for gallbladder HAC. Such data are vital for transitioning from empirical clinical practices towards future precision-medicine strategies tailored to the specific molecular signatures of this rare histological subtype.

The clinical presentation of gallbladder HAC is typically insidious and often indistinguishable from that of conventional GBC or benign biliary diseases (13). Patients frequently present with nonspecific symptoms such as right upper quadrant pain, jaundice, or nausea. However, the ectopic production of AFP may lead to paraneoplastic manifestations or elevated serum AFP levels, necessitating a differential diagnosis that includes metastatic HCC or germ cell tumours (5). The imaging features of HAC often overlap with those of primary GBC, which typically appear as heterogeneous, hypoechoic, or infiltrative masses. Notably, contrast-enhanced ultrasound (CEUS) and CT frequently reveal a characteristic “arterial hyperenhancement and rapid venous washout” pattern (7). This enhancement signature is analogous to that seen in HCC and reflects the tumour’s hypervascular, hepatoid nature (5, 21–23).

Definitive diagnosis of HAC relies on histopathology, characterized by the interweaving of conventional adenocarcinoma components and hepatoid differentiation zones. The latter feature polygonal cells arranged in a trabecular pattern with abundant eosinophilic cytoplasm and prominent nucleoli (9). IHC staining is indispensable, with reported positive rates for AFP and CEA of approximately 70% – 83.3% and 86%, respectively (22, 23). Distinguishing primary gallbladder HAC from metastatic HCC is essential for guiding clinical management (11). While both exhibit similar morphology (21), HAC typically lacks the classic risk factors for HCC, such as cirrhosis (24). Pathologically, the presence of tubular or papillary adenocarcinoma structures is a hallmark of HAC, features that are typically absent in pure HCC. Consequently, the integration of clinical data, serum markers, and a comprehensive IHC profile remains the gold standard for achieving an accurate diagnosis and implementing personalized therapeutic strategies.

Owing to its rarity, the diagnosis of HAC is often delayed, and treatment remains largely empirical. A significant clinical challenge in managing HAC is the current lack of standardized adjuvant protocols. While systemic chemotherapy remains the standard for GBC, we opted for adjunctive TACE in this case. The rationale was based on the tumour’s hepatoid characteristics, including it’s IHC profile and an MRI enhancement pattern analogous to that of HCC. By targeting the hepatic arterial blood supply on which the hepatoid components likely rely, TACE may have effectively eliminated microscopic residual disease or intravascular tumour thrombi, thereby preventing early recurrence. As demonstrated in **Table 1**, the survival of previously reported patients is generally poor, with outcomes ranging from only 1 to 15 months, and many cases result in early death. In contrast, we attributed the patient’s exceptional 68-month survival to several factors. First, the achievement of R0 resection remains the cornerstone of treatment and the strongest predictor of favourable outcomes (5, 12, 13). Second, the tumour exhibited a low mutation burden and microsatellite stability, which may reflect a more genomically stable phenotype than that of other highly aggressive GBCs. Finally, the strategic integration of TACE likely provided a synergistic effect by addressing the specific hepatocellular-like biology of the tumour cells, which may have been less responsive to traditional biliary-type chemotherapy (6, 25).

## Conclusions

This case demonstrates that a multimodal approach, involving radical surgery followed by TACE tailored to hepatoid features, can achieve long-term survival in patients with AFP-producing gallbladder HAC. Future molecular studies are essential to refine these empirical strategies into precision medicine.

## Data Availability

The original contributions presented in the study are included in the article/**Supplementary Material**. Further inquiries can be directed to the corresponding author.
