# Supplementary material for: Long term survival of patients with alpha-fetoprotein-positive hepatoid adenocarcinoma of the gallbladder: a case report and literature review
Source: Front Oncol. 2026 Jun 30;16:1679309. doi: 10.3389/fonc.2026.1679309 (PMC13364591; doi:10.3389/fonc.2026.1679309)
Supplement: Supplementary file 1 [file DataSheet1.docx]

**
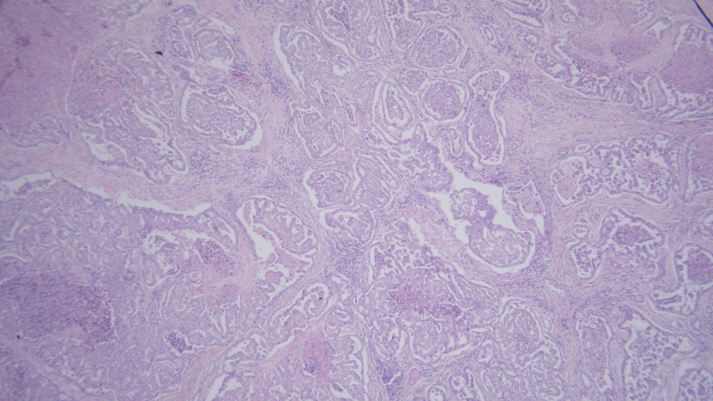
Supplementary figures**

**S1.** **Original picture of H&E staining (×40)**

**
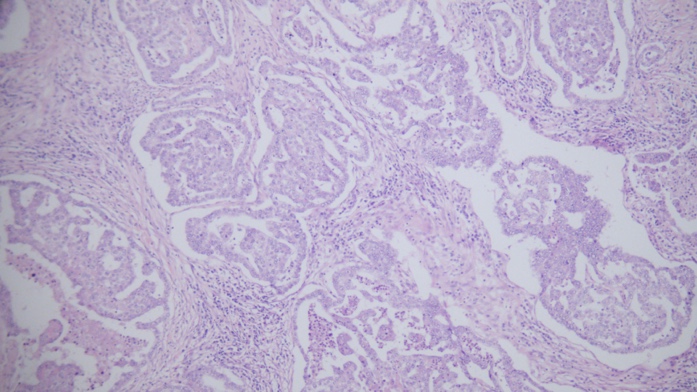
**

**
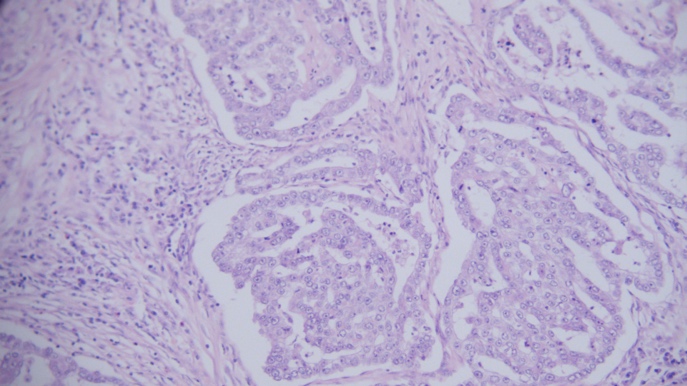
S****2. Original picture of H&E staining (×100)**

**
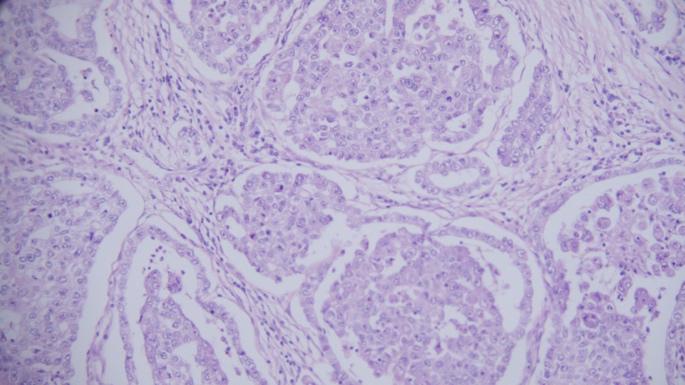
**

**S3. Original picture of H&E staining (×200)**


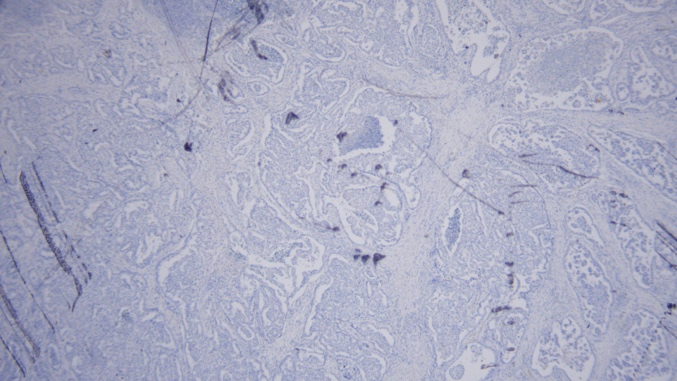

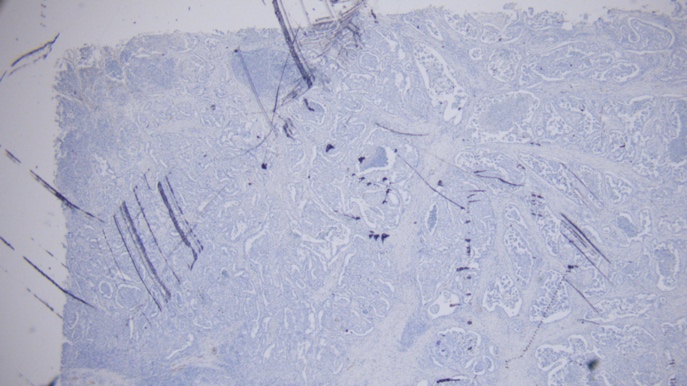


**S****4. Original picture of Hep Par-1 staining (Left×25, Right×40)**


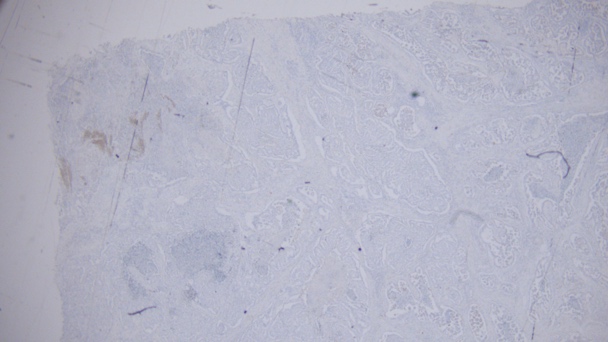
**S5. Original picture of CK-7 staining (Left×25, Right×40)**
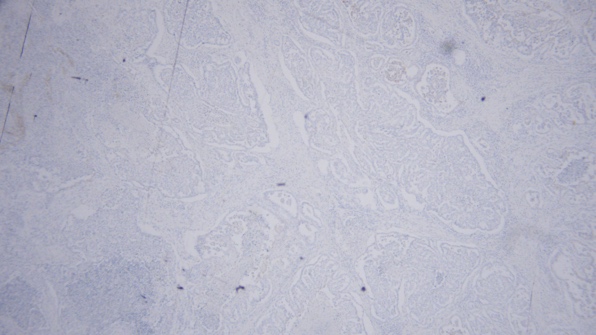


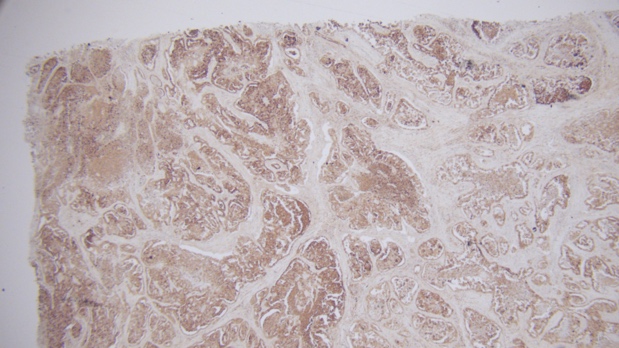

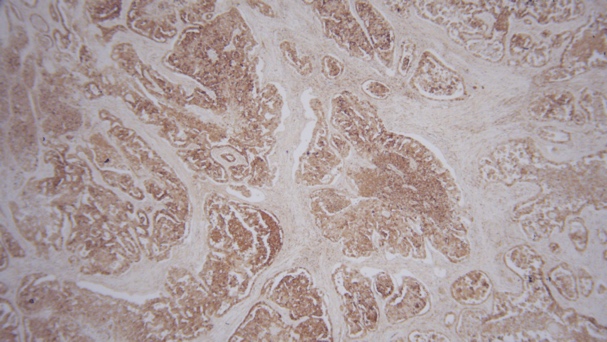


**S6. Original picture of AFP staining (Left×25, Right×40)**


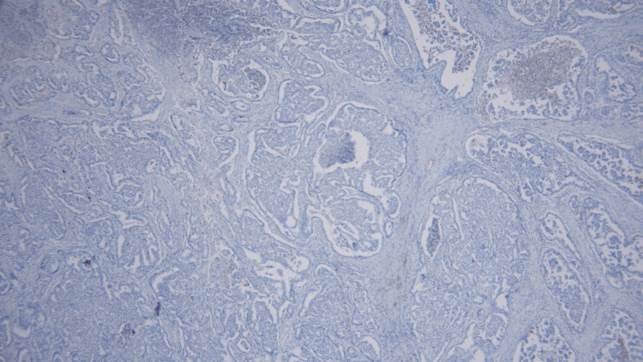

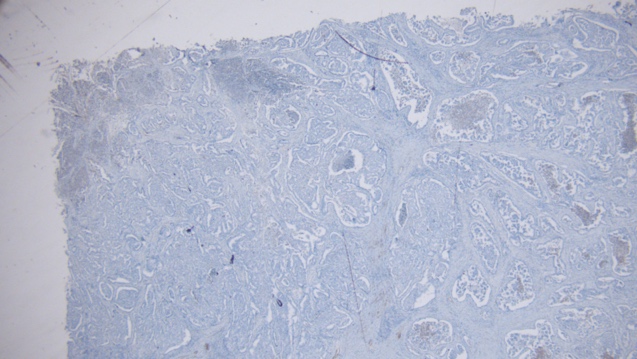


**S7. Original picture of ARG-1 staining (Left×25, Right×40)**


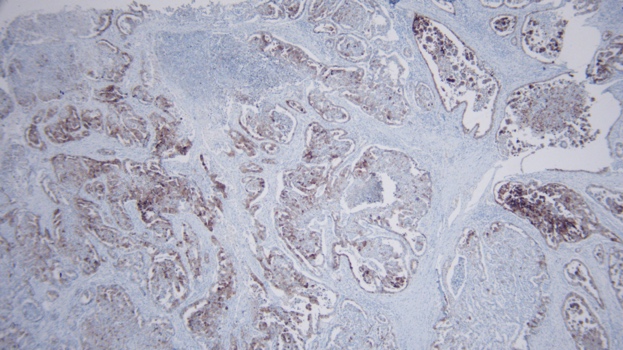

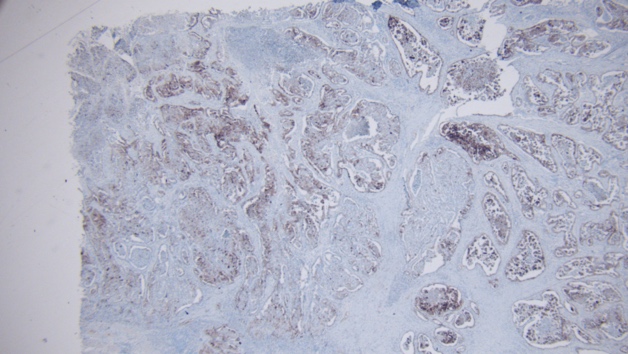


**S8. Original picture of GPC-3 staining (Left×25, Right×40)**


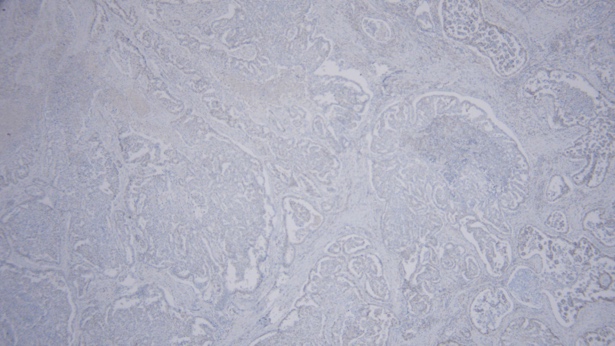

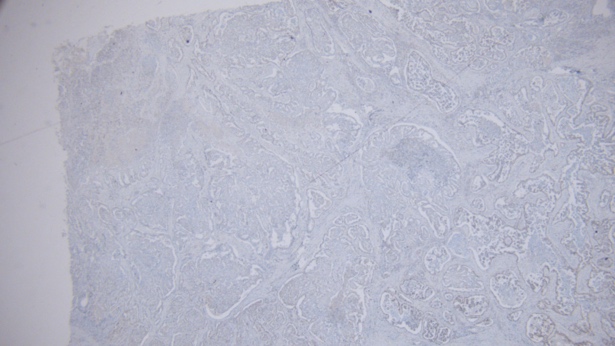
**S9. Original picture of SALL-4 staining (Left×25, Right×40)**
